# FXR-FGF19 signaling in the gut–liver axis is dysregulated in patients with cirrhosis and correlates with impaired intestinal defence

**DOI:** 10.1007/s12072-023-10636-4

**Published:** 2024-02-08

**Authors:** Benedikt Simbrunner, Benedikt S. Hofer, Philipp Schwabl, Kerstin Zinober, Oleksandr Petrenko, Claudia Fuchs, Georg Semmler, Rodrig Marculescu, Mattias Mandorfer, Christian Datz, Michael Trauner, Thomas Reiberger

**Affiliations:** 1 Division of Gastroenterology and Hepatology, Department of Medicine III, Medical University of Vienna, Vienna, Austria; 2 Vienna Hepatic Hemodynamic Laboratory, Division of Gastroenterology and Hepatology, Department of Medicine III, Medical University of Vienna, Vienna, Austria; 3 Christian Doppler Laboratory for Portal Hypertension and Liver Fibrosis, Medical University of Vienna, Vienna, Austria; 4 CeMM Research Center for Molecular Medicine, Austrian Academy of Sciences, Vienna, Austria; 5 Department of Laboratory Medicine, Medical University of Vienna, Vienna, Austria; 6 Department of Internal Medicine, General Hospital Oberndorf, Teaching Hospital, Paracelsus Medical University Salzburg, Salzburg, Austria

**Keywords:** ACLD, Portal hypertension, FXR, FGF19, Intestinal barrier, Gut–liver axis

## Abstract

**Background and aims:**

Experimental studies linked dysfunctional Farnesoid X receptor (FXR)-fibroblast growth factor 19 (FGF19) signaling to liver disease. This study investigated key intersections of the FXR-FGF19 pathway along the gut–liver axis and their link to disease severity in patients with cirrhosis.

**Methods:**

Patients with cirrhosis undergoing hepatic venous pressure gradient measurement (cohort-I *n* = 107, including *n* = 53 with concomitant liver biopsy; *n* = 5 healthy controls) or colonoscopy with ileum biopsy (cohort-II *n* = 37; *n* = 6 controls) were included. Hepatic and intestinal gene expression reflecting FXR activation and intestinal barrier integrity was assessed. Systemic bile acid (BA) and FGF19 levels were measured.

**Results:**

Systemic BA and FGF19 levels correlated significantly (*r* = 0.461; *p* < 0.001) and increased with cirrhosis severity. Hepatic SHP expression decreased in patients with cirrhosis (vs. controls; *p* < 0.001), indicating reduced FXR activation in the liver. Systemic FGF19 (*r* = −0.512, *p* < 0.001) and BA (*r* = −0.487, *p* < 0.001) levels correlated negatively with hepatic CYP7A1, but not SHP or CYP8B1 expression, suggesting impaired feedback signaling in the liver. In the ileum, expression of FXR, SHP and FGF19 decreased in patients with cirrhosis, and interestingly, intestinal FGF19 expression was not linked to systemic FGF19 levels. Intestinal zonula occludens-1, occludin, and alpha-5-defensin expression in the ileum correlated with SHP and decreased in patients with decompensated cirrhosis as compared to controls.

**Conclusions:**

FXR-FGF19 signaling is dysregulated at essential molecular intersections along the gut–liver axis in patients with cirrhosis. Decreased FXR activation in the ileum mucosa was linked to reduced expression of intestinal barrier proteins. These human data call for further mechanistic research on interventions targeting the FXR-FGF19 pathway in patients with cirrhosis.

**Clinical trial number:** NCT03267615

**Graphical abstract:**

Physiology of enterohepatic FXR-FGF19 signaling and its regulation in patients with advanced chronic liver disease (ACLD). (FXR) farnesoid X receptor; (FGF19) fibroblast growth factor 19; (BA) bile acids; (c/dACLD) compensated/decompensated advanced chronic liver disease; (FXR) farnesoid X receptor; (SHP) small heterodimer partner; (OST-α/-β) organic solute transporter subunit alpha/beta; (CYP7A1) cholesterol 7 alpha-hydroxylase; (NTCP) Na+-taurocholate cotransporting polypeptide; (CYP8B1) sterol 12-alpha-hydroxylase; (HVPG) hepatic venous pressure gradient; (TJ) tight junctions; (AMP) antimicrobial peptides; (ASBT) Apical Sodium Dependent Bile Acid Transporter; (ZO 1) zonula occludens-1; (OCLN) occluding; (DEFA5) alpha-5-defensin.
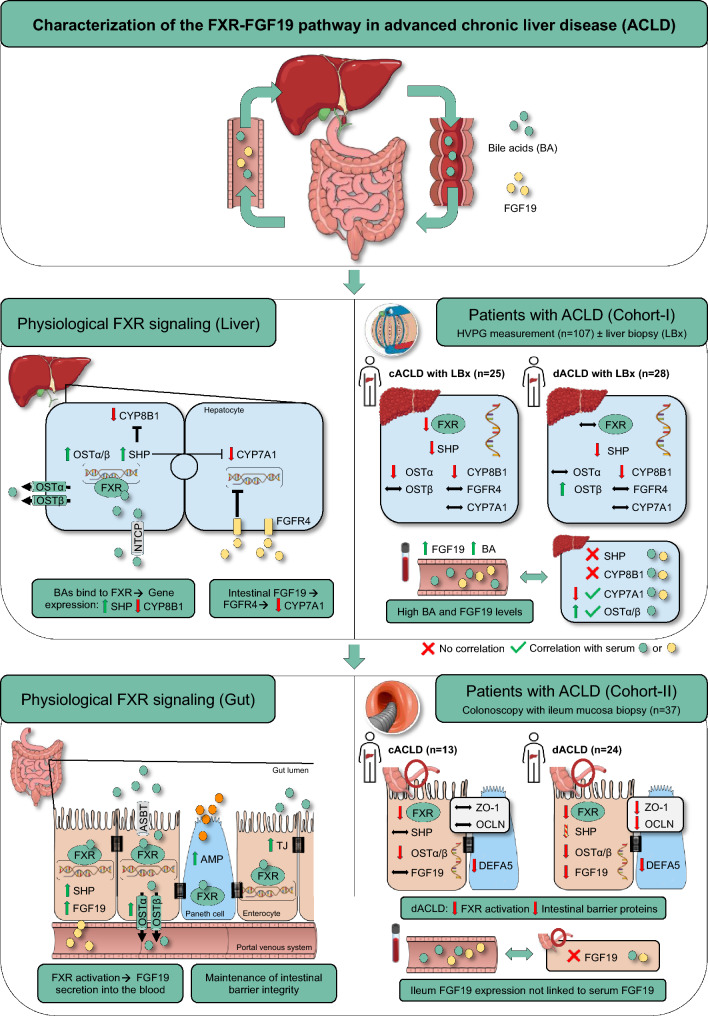

**Supplementary Information:**

The online version contains supplementary material available at 10.1007/s12072-023-10636-4.

## Introduction

Farnesoid X receptor (FXR) is the most important endogenous receptor for bile acids (BA) and highly expressed in the liver and intestines [1]. FXR signaling modulates the synthesis and enterohepatic circulation of BAs, hepatic inflammation and fibrogenesis, and intestinal defence mechanisms protecting against bacterial translocation (BT) from the gut [2]. Activation of FXR in the intestines leads to the intestinal release of fibroblast growth factor-19 (FGF19), which is secreted into the portal vein and leads to suppression of hepatic BA synthesis, thus, acting as an important feedback mechanism [2].

Experimental studies indicate that FXR signaling plays an important mechanistic role in the context of liver cirrhosis. Animal models of chronic liver disease exhibited an impaired intestinal barrier and increased BT in the gut, particularly in the ileum [3–5]. Concordantly, intestinal integrity and BT was ameliorated following the oral administration of BAs or FXR agonists in rodent models of cirrhosis, paralleled by an improvement of BT-associated inflammation and liver damage [4–6]. Furthermore, FXR agonists reduced fibrogenesis and portal hypertension in rodents with cirrhosis [7–9].

However, the state of FXR activation in the liver and intestine of patients with advanced chronic liver disease (ACLD) and its relation to disease severity remains poorly defined. Studies in patients with cholestatic liver disease partially indicated a dysregulation of FXR signaling in liver tissue and intestinal mucosa, however, mostly including patients in earlier (‘pre-cirrhotic’) stages of liver disease or at the timepoint of liver transplantation [10–12]. Considering the pivotal role of BT-induced systemic inflammation, fibrogenesis and portal hypertension for the progression and prognosis of ACLD [13], understanding the association between ACLD severity and FXR signaling in humans is highly relevant to assess the translatability of mechanistic concepts derived from previous experimental studies.

This study aimed to characterize hepatic and intestinal FXR activation in patients with ACLD undergoing (i) hepatic venous pressure gradient (HVPG) measurement with transjugular liver biopsy or (ii) colonoscopy with ileum biopsy. Expression of FXR-induced genes was correlated with disease severity and circulating FGF19 and BA levels. Furthermore, the link between FXR activation and expression of genes associated with intestinal barrier function was assessed.

## Patients and methods

### Study design, patient selection and clinical characterization

Patients with a confirmed diagnosis of ACLD, defined by HVPG ≥ 6 mmHg, liver stiffness ≥ 15 kPa and/or F3/F4 on liver histology, were recruited into the Vienna Cirrhosis Study (VICIS), a prospective observational study registered at ClinicalTrials.gov (NCT03267615) including a biobank for human tissue and body fluids. Patients with previous liver transplantation, non-cirrhotic portal hypertension, hepatocellular carcinoma beyond Milan criteria, or other active malignancies were excluded for this study. All data reported in this study were collected from patients with ACLD managed at the Vienna General Hospital. Patients in Cohort-I (*n* = 107) underwent hepatic vein catheterization for HVPG measurement, of whom *n* = 53 patients had concomitant transjugular liver biopsy (Cohort-Ia) while *n* = 54 patients had no liver biopsy (Cohort-Ib). Patients in Cohort-II (*n* = 37) underwent colonoscopy with an ileum mucosa biopsy (Cohort-II; Supplementary figure 1). Patient cohorts were not essentially overlapping since recruitment into Cohort-I and -II was performed independently.

### Measurement of hepatic venous pressure gradient and liver biopsy

HVPG measurement and transjugular liver biopsy were performed according to a standard operating procedure, as demonstrated previously [14]. Distinct steps of the procedures are outlined in the Supplementary material. Liver tissue control samples for gene expression analyses were sampled from five patients without chronic liver disease undergoing resection of liver tumors at the Medical University of Vienna, Austria included in the prospective LIVERMATRIX study. Absence of chronic liver disease was determined by patient history, pre-surgery imaging and confirmed by histological reports from liver resection specimens.

### Colonoscopy and intestinal mucosa biopsy

Colonoscopy in patients with ACLD was performed in line with a standard operating procedure at the endoscopy unit of the Division of Gastroenterology and Hepatology, Medical University of Vienna, Austria. Mucosa biopsies from the ileum were taken by a standard biopsy forceps. Control ileum biopsies were derived from six liver-healthy individuals undergoing screening colonoscopy at the Department of Internal Medicine, Health Center Oberndorf, Salzburg, Austria, included in the SAKKOPI study. Absence of chronic liver disease was confirmed by patient history, laboratory parameters and liver elastography.

### Processing of tissue, RNA isolation, and gene expression analysis

Liver and ileum specimens were transferred into RNAlater^TM^ stabilization solution (ThermoFisher Scientific, Waltham, Massachusetts, USA). RNA was isolated using a standard TRIzol-based protocol and stored at −80 °C. RNA quality was determined by agarose gel electrophoresis and only samples with intact RNA were further considered for this study. Reverse transcription into cDNA was performed with the High Capacity cDNA Reverse Transcription Kit (Thermo Fisher Scientific, Waltham, MA, USA) in line with the manufacturer’s protocol. Further details are depicted in the Supplementary material.

Gene expression was determined by real-time polymerase chain reaction (RT-PCR) with TaqMan® Universal PCR Master Mix (Thermo Fisher Scientific, Waltham, MA, USA) and commercially available primers (Thermo Fisher Scientific, Waltham, MA, USA), using a 7500 Fast RT-PCR System thermocycler (Applied Biosystems, Foster City, CA, US). Relative gene expression was calculated adapted to the 2^-∆∆Ct^ method [15], determining the logfold expression level of target genes in patients as compared to control samples. The following target genes were assessed: FXR, small heterodimer partner (SHP), organic solute transporter alpha/beta (OST-α/OST-β), FGF19 in Cohort-Ia/II; FGF receptor-4 (FGFR4), CYP7A1, CYP8B1 in Cohort-Ia; zonula occludens-1 (ZO-1), occludin (OCLN), and alpha-5-defensin (DEFA5) in Cohort-II. Primer serial numbers and further details on RT-PCR are delineated in the Supplementary material.

### Biomarker measurements

Biomarkers were measured from samples obtained from the catheter introducer sheath at the timepoint of hepatic vein catheterization (Cohort-I) or from a peripheral vein at the timepoint of colonoscopy (Cohort-II). Standard laboratory markers, total BA, and inflammation parameters were assessed by the ISO-certified Department of Laboratory Medicine, Medical University of Vienna, Vienna, Austria. FGF19 levels were measured by ELISA (R&D Systems Inc., Minneapolis, USA) at the Division of Gastroenterology and Hepatology, Medical University of Vienna, Austria according to the manufacturer’s instructions. Further details are delineated in the Supplementary material.

### Statistical analysis

Statistical calculations were performed with IBM SPSS Statistics 27 (IBM, Armonk, NY, USA) and GraphPad Prism 9 (GraphPad Software, La Jolla, CA, USA). Continuous variables are reported as mean ± standard error of the mean or median with interquartile range (IQR) and categorical variables are displayed as absolute (*n*) and relative (%) frequencies. Student’s *t* test, Mann–Whitney *U* test, Kruskal–Wallis test, or Analysis of variance (ANOVA) were used for comparison of continuous variables. Post-hoc comparison with Tukey’s or Dunn’s multiple comparisons test was performed. Chi squared or Fisher’s Exact test was applied for comparison of categorical variables. Correlation between continuous variables was determined by Spearman correlation coefficients (95% confidence interval). A two-sided *p*-value < 0.05 denoted statistical significance for all analyses.

## Results

### Patient characteristics

Patients undergoing hepatic vein catheterization and transjugular liver biopsy (Cohort-Ia) had a median age of 58 (48–64) years, were predominantly male (*n* = 35, 66%), had a median HVPG of 15 (11–19) mmHg, a median MELD score of 10 (8–14) points, and 28 (53%) patients had decompensated ACLD (dACLD). The most prevalent etiology of ACLD was alcohol-related liver disease (ALD; *n* = 26, 49%). Patients in Cohort-Ib had a significantly higher prevalence of Child-Turcotte-Pugh (CTP) stages B/C (Table 1).

**Table 1:** Patient characteristics

Definition	Cohort-I (*n* = 107) HVPG measurement	Cohort-Ia (*n* = 53) with LBX	Cohort-Ib (*n* = 54) without LBX	Cohort-II (*n* = 37) Colonoscopy + ileum biopsy
Age (years)	57 (50–65)	58 (46–64)	57 (51–66)	58 (51–64)
Sex (M, %)	68 (64)	35 (66)	33 (61)	32 (86)
Etiology (*n*, %)				
ALD	54 (50)	26 (49)	28 (52)	20 (54)
Viral	18 (17)	7 (13)	11 (20)	9 (24)
ALD + Viral	1 (1)	1 (2)	0 (0)	0 (0)
NASH	10 (9)	7 (13)	3 (6)	2 (5)
Cholestatic	1 (1)	1 (2)	0 (0)	0 (0)
Other	23 (21)	11 (21)	12 (22)	6 (16)
dACLD (*n*, %)	71 (66)	28 (53)	43 (79)	24 (64)
HVPG (mmHg)	15 (11–21)	15 (11–19)	18 (10–22)	17 (10–21)^a^
MELD score (points)	11 (8–16)	10 (8–14)	14 (10–18)	11 (10–13)
CTP-stage				
A	48 (45)	30 (58)	18 (33)	18 (49)
B	34 (32)	20 (37)	14 (26)	17 (46)
C	25 (23)	3 (5)	22 (41)	2 (5)
HCC (*n*, %)	4 (4)	4 (7)	0 (0)	2 (5%)
BA (µmol/L)	20.9 (7.60–50.3)	16.9 (6.90–36.2)	25.8 (8.58–68.9)	13.5 (3.00–38.7)
FGF19 (pg/mL)	147 (99–244)	141 (91.2–232)	157 (110–275)	132 (61.3–274)
CRP (mg/dL)	0.32 (0.17–0.76)	0.28 (0.15–0.63)	0.34 (0.20–1.01)	0.38 (0.21–0.82)
IL-6 (pg/mL)	8.77 (4.77–19.3)	8.10 (4.09–15.1)	10.4 (5.09–27.1)	9.03 (5.42–16.1)
PCT (ng/mL)	0.10 (0.07–0.16)	0.11 (0.07–0.14)	0.10 (0.04–0.19)	0.08 (0.04–0.13)
LBP (µg/mL)	7.01 (5.08–8.01)	6.74 (5.27–7.93)	7.31 (5.02–8.49)	7.71 (6.00–10.9)

*ALD* alcohol-related liver disease; *BA* bile acids; *CRP* C-reactive protein; *CTP* Child–Turcotte–Pugh; *HVPG* hepatic venous pressure gradient; *IL-6* interleukin-6; *LBP* lipopolysaccharide binding protein; *LBX* liver biopsy; *M* male sex; *MELD* model of end stage liver disease; *NASH* non-alcoholic steatohepatitis; *PCT* procalcitonin

^a^HVPG available in 28 (76%) patients

Patients undergoing colonoscopy (Cohort-II) had a median age of 58 (51–64) years, were predominantly male (*n* = 32, 86%), displayed a median MELD of 11 (10–13) points, and 24 (64%) were classified as dACLD. ALD (*n* = 20, 54%) and viral hepatitis (*n* = 9, 24%) were the most frequent etiologies of ACLD (Table 1). 28 (76%) patients had HVPG measurement within a median time interval of 4.5 (1.0–16.0) months to colonoscopy and exhibited a median HVPG of 17 (10–21) mmHg.

Characteristics of patients stratified by compensated ACLD (cACLD) and dACLD in Cohort-Ia and Cohort-II are depicted in Supplementary tables S1/S2.

### Bile acid and FGF19 serum levels correlate with disease severity

**Fig. 1:**
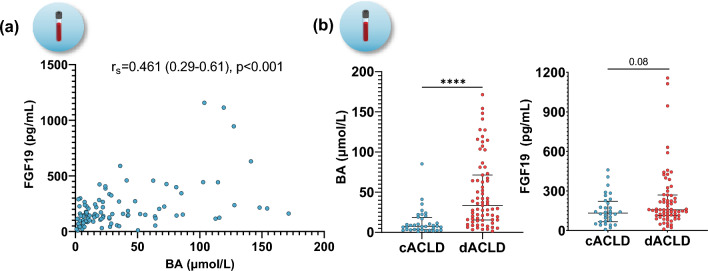
**A** Correlation between bile acid (BA) and fibroblast growth factor-19 (FGF19) serum levels. **B** BA and FGF19 serum levels in patients with compensated and decompensated ACLD. Statistical analysis: Spearman’s correlation coefficient was calculated to assess the association between continuous variables. Mann–Whitney *U* test was applied to compare continuous variables between groups. *BA* bile acid; *FGF19* fibroblast growth factor-19; *c/dACLD* compensated/decompensated advanced chronic liver disease; *HVPG* hepatic venous pressure gradient

BA serum levels displayed a statistically significant correlation with FGF19 serum levels (Spearman’s *r*_s_ = 0.461 [0.29–0.61], *p* < 0.001; Fig. 1A). More than 60% of patients in Cohort-I had BA levels above the upper limit of normal (cACLD: 36%, dACLD: 82%; Supplementary Figure S2). BAs significantly increased in patients stratified by portal hypertension severity (HVPG 6–15 mmHg: 8.98 [5.20–19.4]; HVPG ≥ 16 mmHg: 40.1 [21.5–70.4]; *p* < 0.001) and disease stage (cACLD: 7.55 [3.38–18.6]; dACLD: 33.5 [15.4–71.4]; *p* < 0.001), while FGF19 levels tended to increase in patients with dACLD (cACLD: 133 [67.3–222]; dACLD: 157 [114–270]; *p* = 0.084) but were comparable in patients stratified by HVPG (*p* = 0.195) (Fig. 1B; Supplementary figure S3). Interestingly, the ratio between FGF19 and BA levels was significantly lower in patients with severe portal hypertension (HVPG ≥ 16 mmHg: 4.92 [2.13–8.79] vs. HVPG  < 16 mmHg: 12.8 [8.90–21.8], *p* < 0.001) and dACLD (dACLD: 5.82 [2.84–11.7] vs. cACLD: 14.0 [7.81–21.8], *p* < 0.001), suggesting a relative increase of BA levels as compared to systemic FGF19 levels in patients with severe portal hypertension or dACLD (Supplementary figure S4).

### Hepatic FXR activation in patients with cirrhosis

**Fig. 2:**
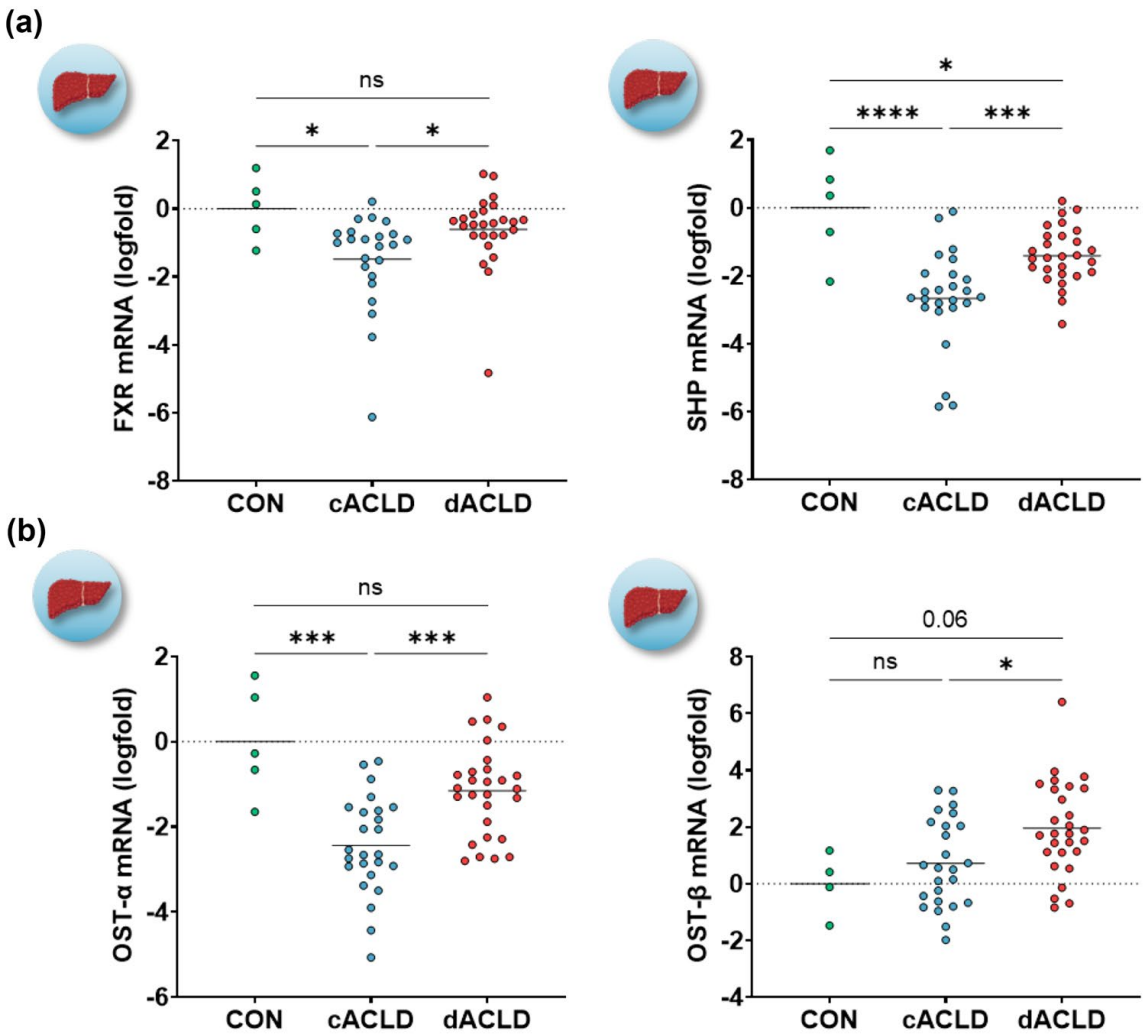
Hepatic expression of FXR and FXR-dependent genes patients with compensated and decompensated ACLD. Statistical analysis: Ordinary one-way analysis of variance (ANOVA) with Tukey’s multiple comparisons test was applied to compare continuous variables between groups. *FXR* farnesoid X receptor; *SHP* small heterodimer partner; *OST-α/-β* organic solute transporter-α/-β; *CON* control group; *c/dACLD* compensated/decompensated advanced chronic liver disease

To determine the state of FXR expression and FXR activation in liver tissue of patients with ACLD, we assessed the expression of FXR and FXR-dependent genes (SHP, OST-α, OST-β). Hepatic FXR expression was decreased in patients with cACLD (logfold −1.49 ± 0.29; *p* = 0.044 vs. controls), while being similar in patients with dACLD, as compared to controls (logfold −0.61 ± 0.21, *p* = 0.565 vs. controls; *p* = 0.039 vs. cACLD). SHP expression in the liver was reduced in cACLD (logfold −2.66 ± 0.29, *p* < 0.001 vs. controls) and dACLD (logfold −1.41 ± 0.16, *p* = 0.045 vs. controls), indicating reduced FXR activation. Nevertheless, SHP expression was significantly higher in patients with dACLD as compared to cACLD (*p* < 0.001; Fig. 2A). Expression of the basolateral BA transporter OST-α was reduced in patients with cACLD (logfold −2.44 ± 0.23, *p* < 0.001 vs. controls), but was statistically similar to controls in patients dACLD (logfold −1.16 ± 0.20, *p* = 0.092 vs. controls). OST-α expression was significantly higher in patients with dACLD compared to cACLD (*p* < 0.001). Finally, hepatic OST-β expression levels were higher in patients with dACLD (logfold 1.97 ± 1.31), than in controls (*p* = 0.059) and patients with cACLD (logfold 0.72 ± 0.31, *p* = 0.016; Fig. 2B; Supplementary Table S4).

### Hepatic FXR-FGF19 feedback signaling and BA synthesis

**Fig. 3:**
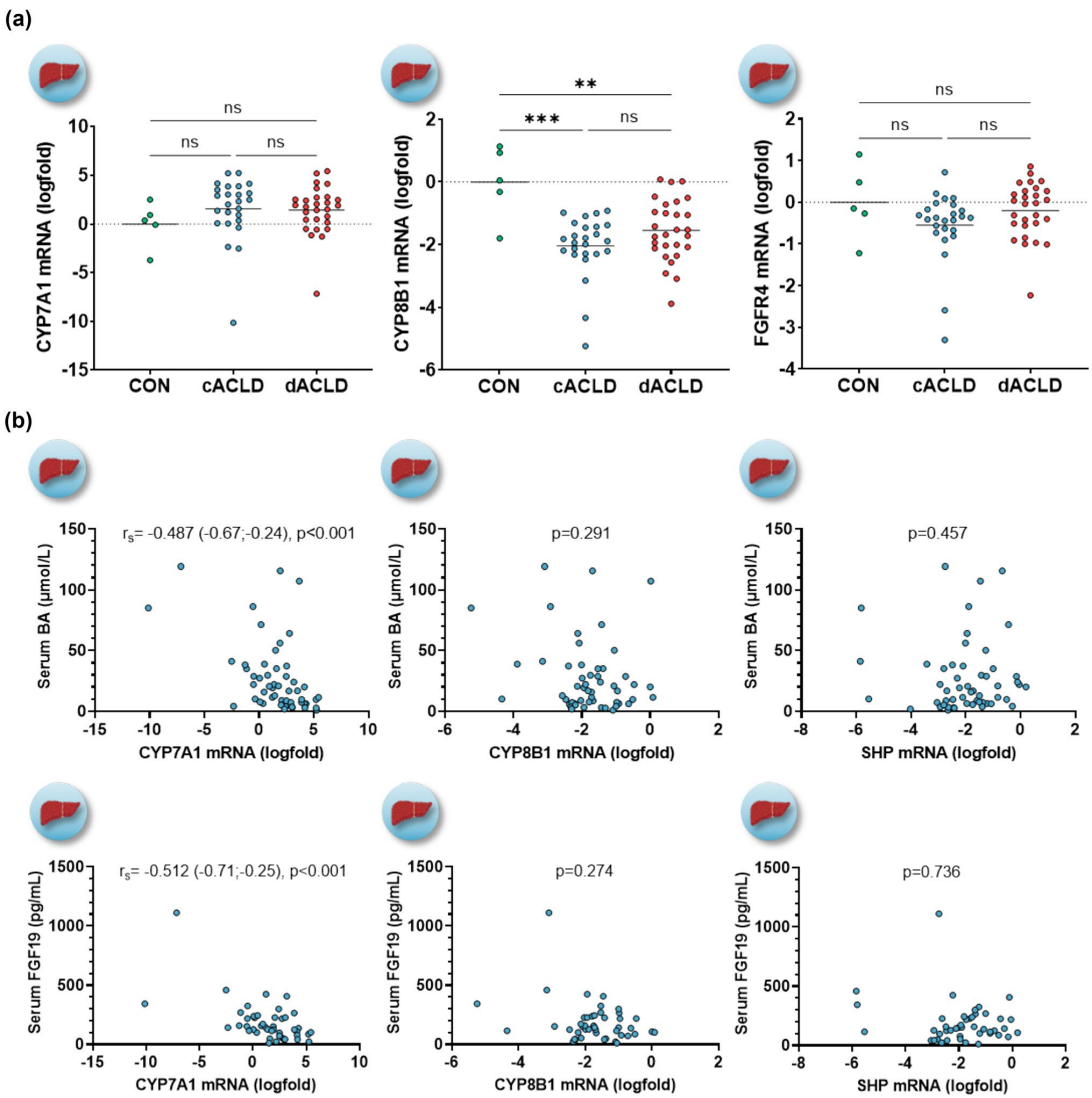
**A** Hepatic expression of FXR-FGF19-regulated genes for bile acid synthesis, and **B** correlation between serum bile acid and FGF19 levels and hepatic expression of FXR-regulated genes in patients with ACLD. Statistical analysis: ordinary one-way analysis of variance (ANOVA) with Tukey’s multiple comparisons test was applied to compare continuous variables between groups. Spearman’s correlation coefficient was calculated to assess the association between continuous variables. *SHP* small heterodimer partner; *FGFR4* fibroblast growth factor receptor-4; *CYP7A1* cholesterol 7 alpha-hydroxylase; *CYP8B1* sterol 12-alpha-hydroxylase; *BA* bile acid; *FGF19* fibroblast growth factor-19

We investigated whether the expression of important genes for hepatic BA synthesis and FGF19-dependent feedback signaling (i.e., CYP7A1, CYP8B1, and FGF receptor 4 [FGFR4]) was linked to disease severity, FXR activation, or FGF19 and BA serum levels in patients undergoing liver biopsy (Cohort-Ia). Expression of CYP7A1 (*p* = 0.514), the main enzyme for de-novo BA synthesis, and FGFR4 (*p* = 0.156), the receptor mediating FGF19-dependent feedback signaling for BA synthesis, displayed no significant difference between controls and patients with ACLD. Conversely, CYP8B1 expression, an enzyme for the alternative pathways regulating the composition of the BA pool, was lower as compared to controls (cACLD: logfold −2.04 ± 0.20, *p* < 0.001; dACLD: logfold −1.55 ± 0.19, *p* < 0.001; Fig. 3A).

Hepatic SHP displayed a significant positive correlation with FXR (*r*_s_ = 0.774, 0.63–0.87, *p* < 0.001), OST-α (*r*_s_ = 0.754, 0.60–0.85, *p* < 0.001), OST-β (*r*_s_ = 0.435, 0.18–0.64, *p* = 0.001), and also an association with CYP8B1 (*r*_s_ = 0.663, 0.47–0.80, *p* < 0.001) and FGFR4 expression (*r*_s_ = 0.437, 0.18–0.64, *p* = 0.001), whereas SHP was not linked to CYP7A1 expression (*r*_s_ = 0.096, *p* = 0.494; Supplementary Figure S5). CYP7A1 expression showed a significant negative correlation with systemic BA (*r*_s_ = −0.487, −0.67–[−]0.24, *p* < 0.001) and FGF19 serum levels (*r*_s_ = −0.512, −0.71–[−]0.25, *p* < 0.001). Conversely, neither systemic BA nor FGF19 levels were linked to hepatic expression of CYP8B1 or SHP (Fig. 3B). Systemic BA exhibited a positive association with hepatic OST-α (*r*_s_ = 0.246, −0.03 to 0.49, *p* = 0.076), and particularly with OST-β expression (*r*_s_ = 0.358, 0.09–0.58, *p* = 0.009; Supplementary Figure S5).

Based on conflicting data from studies whether the liver expresses FGF19 under pathological conditions [2], we evaluated hepatic FGF19 expression in liver biopsies. FGF19 mRNA was only detected in 57% (*n* = 28/49; *n* = 4 not analysed due to limited cDNA availability) of liver biopsies. BA serum levels were significantly higher in patients with detectable hepatic FGF19 mRNA (25.5 [11.6–62.2] vs. 7.30 [4.06–16.2] μmol/L without detectable FGF19 mRNA; *p* = 0.001), while no difference of FGF19 serum levels was noted (*p* = 0.655; Supplementary Figure S6; Supplementary Table S4).

### Intestinal FXR activation, its link to FGF19

**Fig. 4:**
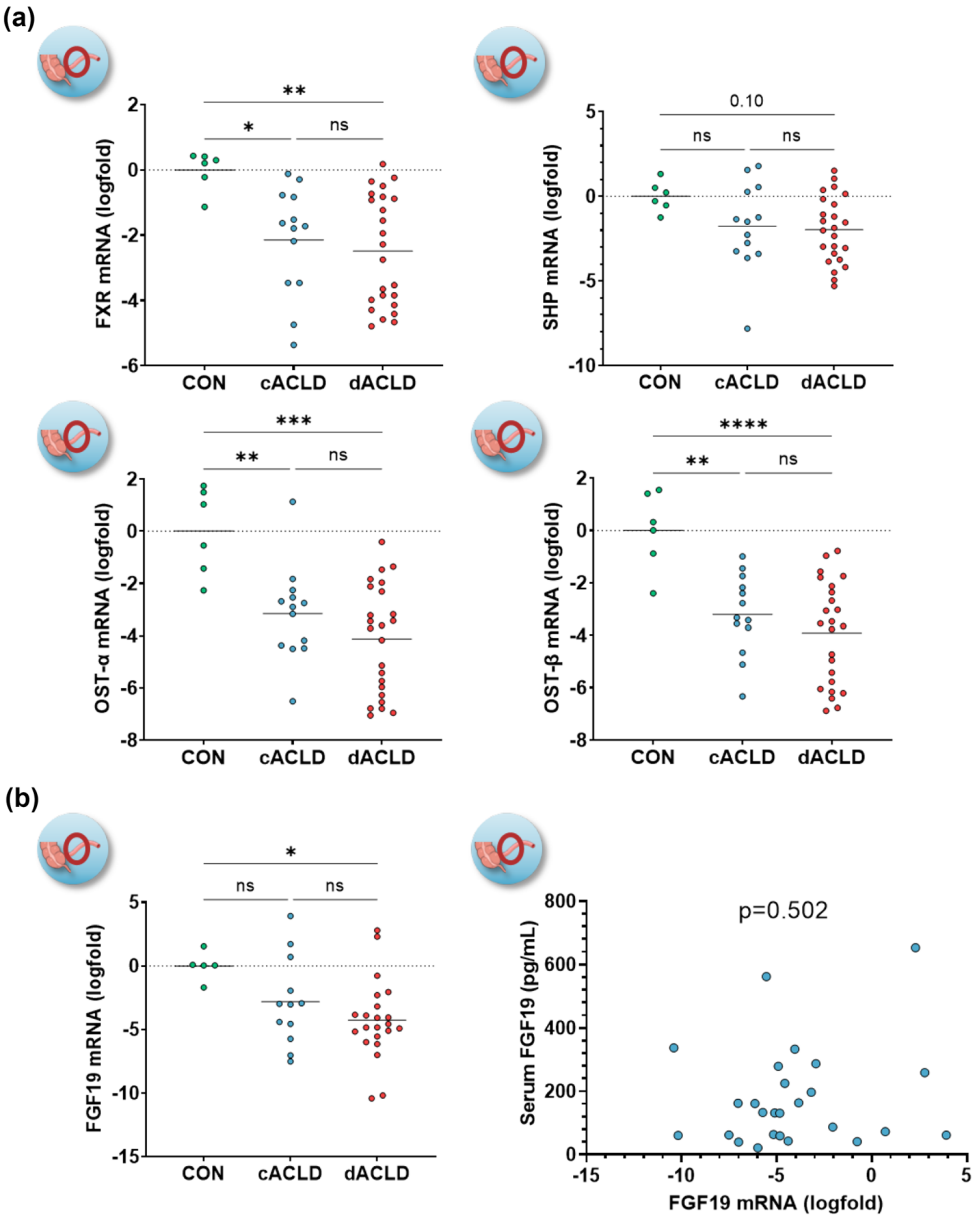
**A** Expression of FXR and FXR-dependent genes in ileum biopsies of patients with ACLD.** B** Expression of FGF19 in ileum biopsies and its correlation with serum FGF19 levels. Statistical analysis: ordinary one-way analysis of variance (ANOVA) with Tukey’s multiple comparisons test was applied to compare continuous variables between groups. Spearman’s correlation coefficient was calculated to assess the association between continuous variables. *SHP* small heterodimer partner; *OST-α/-β* organic solute transporter-α/-β; *BA* bile acid; *FGF19* fibroblast growth factor-19

We investigated whether FXR activation and FGF19 expression in the ileum was linked to disease severity and FGF19 serum levels in patients with ACLD (Cohort-II). FXR expression decreased in patients with cACLD (logfold −2.14 ± 0.46, *p* = 0.025) and dACLD (logfold −2.49 ± 0.35, *p* = 0.004), whereas SHP expression was similar in patients with cACLD (*p* = 0.207) and showed a statistical trend to decrease in dACLD (logfold −1.97 ± 0.40, *p* = 0.107), as compared to controls. Expression of OST-α and OST-β decreased in the ileum mucosa of both patients with cACLD (OST-α: logfold −3.16 ± 0.50, *p* = 0.006; OST-β: logfold −3.21 ± 0.42, *p* = 0.002) and dACLD (OST-α: logfold −4.12 ± 0.42, *p* < 0.001; OST-β: logfold −3.91 ± 0.40, *p* < 0.001) as compared to controls (Fig. 4A). The expression of SHP correlated with FXR (*r*_s_ = 0.864, 0.75–0.93, *p* < 0.001), OST-α (*r*_s_ = 0.825, 0.68–0.91, *p* < 0.001), OST-β (*r*_s_ = 0.885, 0.78–0.94, *p* < 0.001), and with FGF19 (*r*_s_ = 0.602, 0.32–0.79, *p* < 0.001) in the ileum (Supplementary Figure S7).

FGF19 expression was similar between controls and patients with cACLD (*p* = 0.216), interestingly however, it decreased in patients with dACLD (logfold −4.26 ± 0.67, *p* = 0.023). Of note, FGF19 mRNA was not detected in three patients (8%; *n* = 1 cACLD, *n* = 2 dACLD). No FGF19 gene expression in the ileum mucosa was neither linked to serum FGF19 levels (*p* = 0.502) nor serum BA levels (*p* = 0.170). Notably, intestinal FGF19 expression showed a significant negative correlation with MELD (*r*_s_ = −0.447, −0.69 to −0.12, *p* = 0.008; Fig. 4B; Supplementary Figure S8; Supplementary Table S4).

### Intestinal FXR signaling and mucosal defence in cirrhosis

**Fig. 5:**
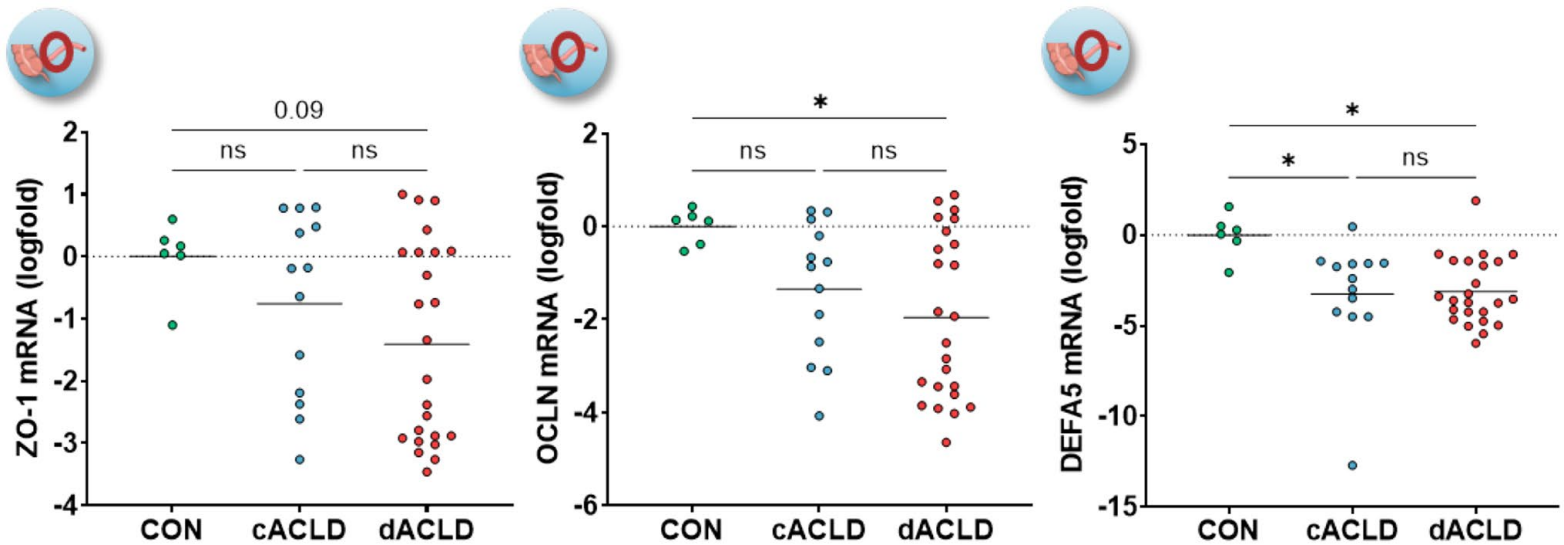
Expression of genes related to intestinal barrier and mucosal defence in the ileum of patients with ACLD. Statistical analysis: ordinary one-way analysis of variance (ANOVA) with Tukey’s multiple comparisons test was applied to compare continuous variables between groups. *ZO-1* zonula occludens-1; *OCLN* occludin; *DEFA5* α5-defensin; *CON* control group; *c/dACLD* compensated/decompensated advanced chronic liver disease

Since FXR signaling regulates intestinal barrier integrity, which is believed to be impaired in the setting of cirrhosis [2], we determined whether FXR activation in the ileum was associated with the expression of genes involved in the mucosal barrier in patients with ACLD. Intestinal expression of FXR and SHP was linked to the expression of the tight junction proteins zonula occludens-1 (ZO-1; FXR: *r*_s_ = 0.836; SHP: *r*_s_ = 0.830) and occludin (OCLN; FXR: *r*_s_ = 0.896; SHP: *r*_s_ = 0.826), and the antimicrobial peptide alpha-5-defensin (DEFA5; FXR: *r*_s_ = 0.820; SHP: *r*_s_ = 0.691; all *p* < 0.001; Supplementary figure S7). Importantly, the expression of ZO-1 and OCLN decreased in patients with dACLD (ZO-1: logfold −1.41 ± 0.32, *p* = 0.099; controls vs. ACLD *p* = 0.07; OCLN: logfold −1.96 ± 0.36, *p* = 0.025; controls vs. ACLD *p* < 0.05), while DEFA5 expression was significantly lower in both patients with cACLD and dACLD as compared to controls (cACLD: logfold −3.25 ± 0.88, *p* = 0.017, dACLD: logfold −3.11 ± 0.38, *p* = 0.013; controls vs. ACLD *p* < 0.01; Fig. 5; Supplementary Table S4).

## Discussion

The present study characterized hepatic and intestinal FXR signaling, circulating BA and FGF19, and disease severity in patients with ACLD. BA and FGF19 serum levels were assessed in 107 patients undergoing HVPG measurement. In a subgroup of 53 patients with concomitant transjugular liver biopsy, expression of FXR and genes reflecting FXR activation were assessed and compared with circulating biomarkers. Furthermore, the relation between FXR activation and the expression of genes related to mucosal defence and inflammation were assessed in ileum biopsies from 37 patients undergoing colonoscopy.

BA and FGF19 serum levels exhibited a direct correlation and increased in patients with dACLD. More than half of patients displayed elevated serum BA levels, which is in line with previous studies in patients with (A)CLD that reported a link between serum BA [16–18] or FGF19 levels [12, 19] and disease severity. Similarly, a correlation between BA and FGF19 levels was also reported in patients with primary biliary cholangitis (PBC) [20], suggestive towards a regulatory mechanism to suppress BA synthesis. Interestingly, the FGF19:BA ratio decreased in patients with dACLD, which might indicate a further increment of dysfunctional feedback regulation in this disease stage. Although it must be acknowledged that our study does not report concentrations from portal venous blood, BA levels in peripheral blood are considered to mirror portal venous blood levels in cirrhosis—likely due to portosystemic shunting [21–23]. The link between BA serum levels and disease severity already indicates that FXR-FGF19 signaling is impaired in ACLD. To this end, our study aimed to characterize key molecular intersections of this pathway along the gut–liver axis using patient-derived hepatic and intestinal tissue specimens.

Hepatic SHP expression was reduced in patients with both compensated and decompensated ACLD indicating impaired FXR activation [24]. In patients with ACLD undergoing colonoscopy with ileum biopsy, FXR expression significantly decreased as compared to controls, while SHP tended to decrease in patients with dACLD. Concordantly, the expression of the basolateral BA efflux pumps OST-α and OST-β—that are induced upon activation of FXR [25–27]—were strongly connected to hepatic and intestinal FXR/SHP expression. Since impairment of hepatic and intestinal FXR signaling is considered to influence numerous pathophysiological processes in chronic liver disease [28], it is tempting to hypothesize that the downregulation of FXR activation in patients with ACLD may provide an explanation for the therapeutic efficacy of FXR agonists observed in previous experimental studies using animal models of chronic liver disease [7–9, 29], and in patients with cholestatic and metabolic liver disease [2]. Nevertheless, a closer look on our data and previous studies is warranted.

In the present study, patients with dACLD exhibited increased expression of FXR and SHP as compared to patients with cACLD. Considering that hepatic FXR activation is believed to ameliorate liver disease [30], it is surprising that patients with decompensated cirrhosis—having a worse prognosis than patients with cACLD [31]—showed higher expression levels of FXR and SHP. Performing direct comparisons to the results of animal studies investigating FXR agonists are difficult since these models mostly resemble cACLD (or even pre-cirrhotic stages). Experimental studies using cholestatic, toxic, or metabolic liver disease animal models to investigate the effects of FXR agonists reported a similar expression of SHP in untreated diseased animals as compared to healthy animals, with pharmacological FXR activation causing a subsequent overexpression of hepatic SHP [8, 9, 29]. Conversely, liver biopsies in our study were obtained in rather stable patients while earlier studies in humans mostly obtained liver tissue specimens at the timepoint of liver transplantation—therefore likely representing distinct patient collectives—and reported highly heterogenous results [10–12, 32, 33]. At this point, we can only speculate on the significant upregulation of FXR, SHP, and OSTα/OSTβ in patients with dACLD as compared to cACLD patients. Since patients with dACLD also displayed higher BA levels as compared to patients with cACLD, this observation might be related to a persistent presence of a high BA load in dACLD but could also be related to distinct differences of the hepatic macro- and microenvironment between patients with cACLD and dACLD that leads to a differential regulation of FXR signaling in the liver tissue. This question should be addressed in future studies using novel techniques such as single-cell sequencing.

Importantly, our data suggest that FXR-FGF19-associated regulation of hepatic BA synthesis may be dysfunctional in patients with ACLD. According to physiological concepts of BA homeostasis, the presence of high BA levels should lead to an increase of hepatic SHP expression and suppression of CYP8B1 (determining the composition of the primary BA pool) and partially CYP7A1 (main enzyme for de-novo BA synthesis). Furthermore, elevated serum FGF19 should lead to a suppression of CYP7A1 in the liver *via* FGFR4 [2, 28]. In our study cohort, hepatic CYP7A1 (and FGFR4) expression was similar, while CYP8B1 expression significantly decreased as compared to controls, which is unexpected due to the observed hepatic SHP downregulation in ACLD. Notably, Milkiewicz et al. also reported unchanged gene expression of hepatic CYP7A1, as well as reduced hepatic CYP8B1 expression in patients with PSC as compared to controls [11]. Furthermore, McCormick et al. found a marked decrease of cholic acid synthesis in patients with cirrhosis, which is dependent on CYP8B1 processing [34].

FGF19 and BA levels in the systemic circulation exhibited a significant negative correlation with hepatic CYP7A1 expression, which would seem in line with physiological FGF19-mediated feedback regulation at the first glance. Considering the high prevalence of elevated BA levels in the circulation of patients with ACLD, however, it might be argued that (down-)regulation of BA synthesis is still insufficient. The absence of any association between BA levels and hepatic FXR activation, as well as the positive correlation between hepatic SHP and CYP7A1/CYP8B1 expression (as SHP is rather expected to suppress these genes) further indicates that hepatic FXR-FGF19 feedback signaling is dysfunctional in patients with ACLD. Notably, hepatic OST-β was upregulated (correlating with systemic BA levels), and OST-α expression was significantly higher in patients with dACLD as compared to cACLD, whereas these transporters were downregulated in ileum biopsies. The divergent pattern of the observed down- and upregulation of OST-α/OST-β in intestinal and liver biopsies, respectively, may be explained by the different functional roles on either intestinal BA reabsorption versus hepatocellular elimination of intracellular BAs, as discussed by Boyer et al. in the context of PBC [32]. We acknowledge that our study could not assess protein expression of OST-α/-β due to limited available tissue material owing to the small biopsy size, which represents a limitation of our study.

The ileum exhibits the highest expression levels of FXR along the gastrointestinal tract, and thus, is considered the most relevant intestinal segment involved in FXR-FGF19 signaling [35, 36]. Reduced intestinal FXR activation was reported in rats with CCl_4_-induced cirrhosis (with ascites) and bile-duct ligated (BDL) mice after 5 days (i.e. a non-cirrhotic stage) [4, 35]. In our study cohort, FGF19 expression in the ileum decreased in patients with dACLD, as compared to controls, being linked to lower expression of FXR and SHP in ileum biopsies. No direct correlation between intestinal FGF19 expression and systemic FGF19 levels was observed, indicating that serum FGF19 levels do not essentially reflect intestinal FGF19 expression. These results raise the question whether the ileum is really the only (or main) source of FGF19 in patients with ACLD, since our data do not suggest that high systemic FGF19 levels are related to FXR activation in the ileum in patients with dACLD. In contrast to mice, however, where FGF15 (the mouse orthologue of human FGF19) is absent in the liver [37], the human liver may show increased FGF19 expression under disease conditions [12, 38, 39]. Concordantly, we detected FGF19 mRNA in about 50% of liver biopsies that were linked to high systemic BA (but not FGF19) serum levels. This observation suggests that hepatic FGF19 expression may represent a potential adaption to counteract the increased systemic BA load in ACLD.

We acknowledge that our study cannot elucidate the exact molecular mechanisms disrupting the gut–liver FXR-FGF19 signaling axis in ACLD, but importantly, our results clearly call for a critical assessment of FXR-FGF19-related interventions in the specific setting of ACLD—while therapeutic studies are ongoing in patients with liver disease [28]. In this context it is important to note that safety issues for the clinical use of the steroidal FXR agonist obeticholic acid in patients with ACLD [40, 41] have been reported, and the efficacy of non-steroidal FXR agonists in ACLD remains to be investigated. To this end, randomized clinical trials assessing the efficacy of FXR agonists in humans should include readouts on FXR target engagement from paired liver biopsies or even intestinal biopsies in the study design.

Finally, multiple experimental studies in animal models of cirrhosis have reported increased bacterial translocation and indicated that treatment with both natural and synthetic FXR agonists improved intestinal barrier integrity [3–6, 42]. Since bacterial translocation is considered to promote systemic inflammation and disease progression in ACLD [43–48], translating mechanistic concepts on the relationship between FXR activation and intestinal barrier integrity may hold highly relevant information for the identification of treatment targets. This consideration is particularly relevant since dysregulation of FXR signaling in the cirrhotic liver and altered BA synthesis can affect FXR signaling in the intestines, leading to impaired barrier integrity and bacterial translocation and their detrimental effects on the liver and vice versa [2, 49]. This bidirectional relationship could likely fuel a vicious circle that promotes further deterioration of FXR signaling and progressive dysfunction of the gut–liver axis. Importantly, the intestinal gene expression of the tight junction proteins ZO-1 and OCLN decreased in patients with dACLD and DEFA5 expression was significantly decreased in patients with cACLD and dACLD. While there was a clear trend towards a stepwise decrease of tight junction expression between controls, cACLD, and dACLD, the subgroup comparison between cACLD and dACLD failed to reach statistical significance—which may be explained due to sample size limitations and correction for multiple testing. Nevertheless, since experimental studies in animals pointed towards the ileum as the main gastrointestinal segments relevant for bacterial translocation [50–52], we believe that our data fill an important translational gap on the intestinal barrier and mucosal defence in patients with ACLD, that should be considered for future efforts to therapeutically target bacterial translocation in cirrhosis.

In summary, we found that hepatic and intestinal FXR-FGF19 signaling is dysregulated in patients with cACLD and dACLD which is linked to impaired BA homeostasis. FXR signaling was linked to the expression of intestinal barrier integrity genes, especially in patients with dACLD. These data should be considered for ongoing and future efforts aiming to therapeutically engage FXR-FGF19 signaling and reduce bacterial translocation in ACLD.

### Supplementary Information

Below is the link to the electronic supplementary material.

Supplementary file1 (DOCX 964 KB)

## Data Availability

Data are available at reasonable request to the corresponding author.

## Abbreviations

[c/d]ACLD: [Compensated/decompensated] advanced chronic liver disease
ALD: Alcohol-related liver disease
BA: Bile acids
BDL: Bile-duct ligated
BT: Bacterial translocation
CYP7A1: Cytochrome P450 Family 7 Subfamily A Member 1
CYP8B1: Cytochrome P450 Family 8 Subfamily B Member 1
DEFA5: Alpha-5-defensin
FGF19: Fibroblast growth factor-19
FGFR4: FGF receptor-4
FXR: Farnesoid X receptor
HVPG: Hepatic venous pressure gradient
MELD: Model for End-stage Liver Disease
OCLN: Occludin
OST-α/OST-β: Organic solute transporter alpha/beta
PBC: Primary biliary cholangitis
RT-PCR: Real-time polymerase chain reaction
SHP: Small heterodimer partner
ZO-1: Zonula occludens-1
