# Supplementary material for: FXR-FGF19 signaling in the gut–liver axis is dysregulated in patients with cirrhosis and correlates with impaired intestinal defence
Source: Hepatol Int. 2024 Feb 8;18(3):929–42. doi: 10.1007/s12072-023-10636-4 (PMC11126514; doi:10.1007/s12072-023-10636-4)
Supplement: Supplementary file 1 — Supplementary file1 (DOCX 964 KB) [file 12072_2023_10636_MOESM1_ESM.docx]

**SUPPLEMENTARY MATERIAL**

**Title:** **FXR-FGF19 signaling in the gut-liver axis is dysregulated in patients with cirrhosis and correlates with impaired intestinal defense**

**Table of contents**

[Supplementary methods 2](#_Toc151671972)

[Hepatic venous pressure gradient measurement and transjugular liver biopsy 2](#_Toc151671973)

[Fibroblast growth factor-19 measurement 2](#_Toc151671974)

[RNA isolation 2](#_Toc151671975)

[RNA quality assessment 3](#_Toc151671976)

[Reverse transcription 3](#_Toc151671977)

[Gene expression analysis 3](#_Toc151671978)

[Missing values 4](#_Toc151671979)

[Supplementary figures 5](#_Toc151671980)

[Supplementary tables 11](#_Toc151671981)

[References (used in the supplementary material) 16](#_Toc151671982)

# Supplementary methods

## Hepatic venous pressure gradient measurement and transjugular liver biopsy

Hepatic venous pressure gradient (HVPG) was measured in fasting condition, according to a standard operating procedure published previously [1]. After injection of local anaesthesia, a catheter introducer sheath was inserted under ultrasound guidance in the right internal jugular vein by Seldinger technique. Next, a balloon catheter was introduced through the sheath and advanced into a large hepatic vein under fluoroscopic guidance. Correct position of the catheter tip was confirmed by injecting contrast media while the balloon was inflated, thus, blocking outflow of the contrast agent into the inferior vena cava. After a minimum of three measurements, HVPG was determined as the difference of free and wedged hepatic vein pressure. Transjugular biopsy was performed in the same session, i.e., after HVPG measurement. A biopsy needle introducer sheath was advanced into a hepatic vein under fluoroscopic guidance, followed by cautious introduction of the biopsy needle. Either aspiration or core biopsy was performed [1].

## Fibroblast growth factor-19 measurement

Fibroblast growth factor-19 (FGF19) was measured using the Human FGF-19 Quantikine ELISA kit (R&D Systems Inc., Minneapolis, USA). Duplicate measurements were performed in human serum samples stored at -20°C. Briefly, 100 µL of assay diluent and 100 µL of standard, control, or undiluted serum were added to each well. After four repeats of aspiration and washing, 200 µL of FGF19 conjugate were added. After an incubation period of two hours at room temperature (RT) followed by four repeats of aspiration and washing, 200 µL of substrate solution were added. After incubation for 30 minutes at RT, 50 µL of stop solution were added. Optical density at 450 nm (wavelength correction 570 nm) was determined using an ASYS UVM 340 microplate reader (Biochrom Ltd, Cambridge, UK).

## RNA isolation

RNA was isolated from liver and ileum specimens using a standard TRIzol-based isolation protocol. Briefly, 1 mL TRIzol® (Thermo Fisher Scientific Waltham, MA, USA) was added to the tissue, followed by homogenization at 6500 U/min for 2x20 seconds. After short centrifugation (6000 rpm) and removal of the supernatant, 200 µL chloroform were added, followed by manual shaking for 15 s, incubation at RT for 3 minutes, and centrifugation at 4°C for 10 minutes (10500 rpm). After removal of the supernatant, 1 mL 75% ethanol (-20°C; with nuclease-free water [NFW]) was added, followed by centrifugation at 4°C for 5 minutes (8500 rpm). The supernatant was removed, and the pellet was incubated and dried at 37°C for 15 minutes. Subsequently, 80-100 µL NFW was added, and the sample was incubated in the Thermomixer® comfort (Eppendorf AG, Hamburg, Germany) at 55°C for 10 minutes. RNA concentration was determined by a NanoDrop^TM^ spectrophotometer (ThermoFisher Scientific, Waltham, Massachusetts, USA). RNA was stored at -80°C.

## RNA quality assessment

RNA quality was assessed by agarose gel electrophoresis. Briefly, 5x Tris-Borate-EDTA (TBE) buffer was diluted with aqua bidest. Agarose LE (Biozym Scientific GmbH, Hessisch Oldendorf, Germany) was added to 1x TBE buffer to a final concentration of 1.5%. Subsequently, GelRed® Nucleic Acid Gel Stain (Biotium, Fremont, CA, USA) was added. Loading buffer was composed from 500 µL glycerol, 400 µL 0.5% bromophenol blue, and 100 µL 1x TBE buffer. Loading buffer and RNA or Century™-Plus RNA Markers (ThermoFisher Scientific, Waltham, Massachusetts, USA) were mixed and incubated in a heating block at 80°C for 5 minutes, transferred to ice, spinned down, and loaded onto the plate with agarose gel. Electrophoresis was performed for a duration of 90-120 minutes at 90 volts and analyzed on a UV transilluminator. Samples showing distinct 18S and 28S bands were classified as intact.

## Reverse transcription

Reverse transcription from RNA into cDNA was performed with the High Capacity cDNA Reverse Transcription Kit (Thermo Fisher Scientific, Waltham, MA, USA), according to the manufacturer’s protocol. Reverse transcription mastermix, RNA, and NFW were mixed and incubated for 10 minutes at room temperature, and then transferred to the Thermomixer for two hours at 37°C. NFW was added in the respective concentration to yield a cDNA concentration of 20 ng/µL. cDNA was stored in a -20°C freezer.

## Gene expression analysis

Gene expression was assessed by RT-PCR using TaqMan gene expression kits (TaqMan® Universal PCR Master Mix; Thermo Fisher Scientific, Waltham, MA, USA) and commercially available primers (Thermo Fisher Scientific, Waltham, MA, USA). Primer serial numbers are summarized in **Supplementary Table-S3.**  Briefly, 1 µL (i.e., 20 ng) cDNA, 10.25 µL NFW, 12.5 µL TaqMan® Master Mix, and 1.25 µL primer were pipetted into each well. Triplicate measurements were performed; however, duplicate measurements were performed in case of limited availability of cDNA. RT-PCR was performed using a 7500 Fast RT-PCR System thermocycler (Applied Biosystems, Foster City, CA, US). Polymerase was activated at a temperature of 95°C for 10 minutes, followed by 40 cycles of denaturation (95°C, 15 s) and extension (60°C, 1 min). The detection threshold was manually set at 0.5 for all analyses.

Gene expression was determined following the 2^–∆∆Ct^ method [2]. Beta-2-microglobulin (B2M) served as the housekeeping gene. Next, the difference between the Ct-value of B2M and the Ct-value for each target gene was calculated (∆Ct), followed by calculation of the average ∆Ct-value of controls, and subsequently, the ∆∆Ct value. Finally, the 2^–∆∆Ct^ value was calculated: $2^{-\Delta\Delta Ct}=\frac{2^{-\Delta Ct \left( \mathrm{patient} \right)}}{2^{-mean of \Delta Ct (controls)}}$. For liver controls, n=1 value for OST-β were excluded, after outlier elimination using the ROUT method (Q=0.1%). Expression levels are reported as 2^log^(2^–∆∆Ct^) values.

## Missing values

Missing laboratory or gene expression values arose in some patients undergoing a liver or ileum biopsy (Cohort-Ia and Cohort-II) due to limited amount of tissue or blood samples for scientific purposes. While samples with insufficient RNA quality or minimal amounts of RNA were excluded *a priori* for this study, patients in whom most parameters of interest could be assessed were included, considering the value of (even just partial) readouts from patient tissue and the lack of ethical justification to repeat biopsies solely for scientific use. Similarly, blood specimens were in some cases not available/sufficient to perform the complete biomarker panel for this study. Missing values are indicated in **Supplementary Table-S4**.

# Supplementary figures

**Supplementary Figure-S1. Graphical summary of the study design.**

Abbreviations: (HVPG) hepatic venous pressure gradient; (FGF19) fibroblast growth factor-19; (FXR) farnesoid X receptor.

**Supplementary Figure-S2. Frequency of elevated bile acid serum levels in patients with advanced chronic liver disease (Cohort-I).**


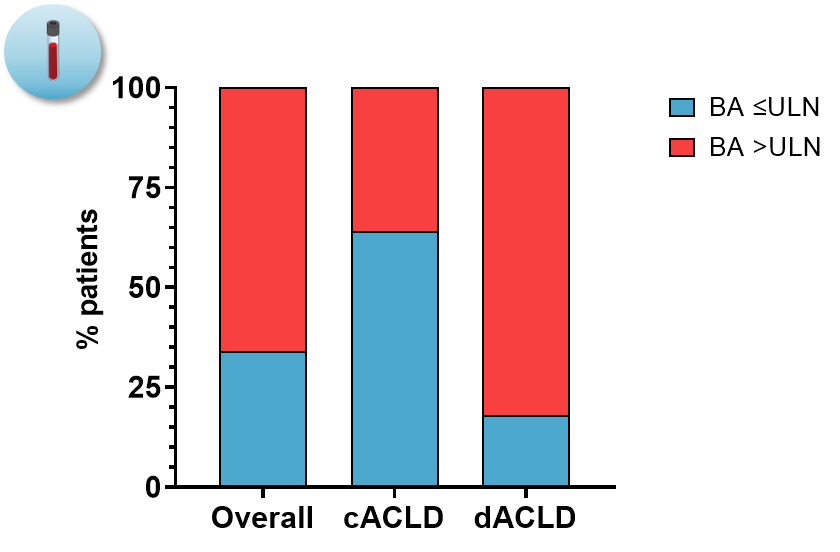


Abbreviations: (FGF19) Fibroblast growth factor-19; (BA) bile acid; (c/dACLD) compensated/decompensated advanced chronic liver disease; (ULN) upper limit of normal

**Supplementary Figure-S3. BA and FGF19 serum levels in patients stratified by hepatic venous pressure gradient (HVPG).**

Statistical analysis: Group comparisons were performed by Mann-Whitney U test. Abbreviations: (FGF19) Fibroblast growth factor-19; (BA) bile acid; (HVPG) hepatic venous pressure gradient

**Supplementary Figure-S4. Fibroblast growth factor-19(FGF19)-to-bile acid(BA) level ratio in patients stratified by disease severity and hepatic venous pressure gradient.**


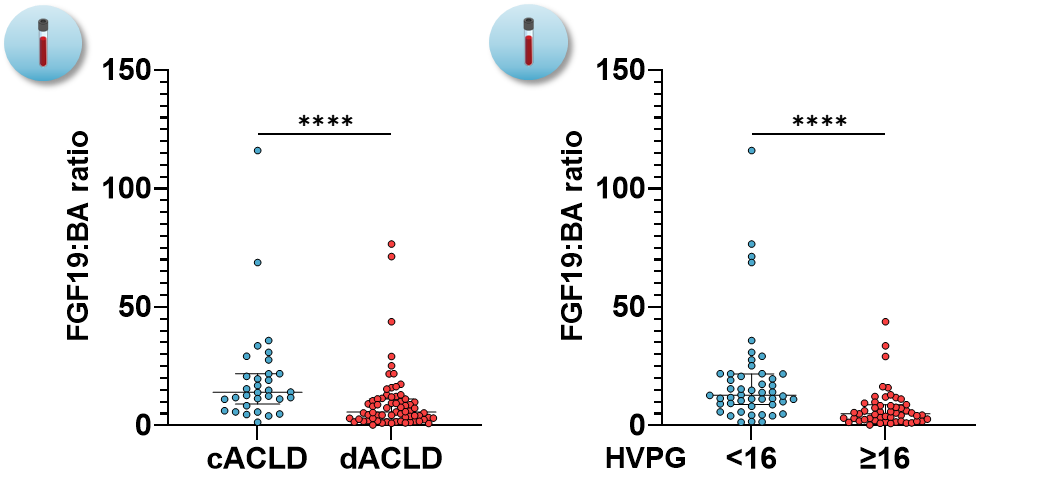


Statistical analysis: Group comparisons were performed by Mann-Whitney U test. Abbreviations: (FGF19) Fibroblast growth factor-19; (BA) bile acid; (c/dACLD) compensated/decompensated advanced chronic liver disease; (HVPG) hepatic venous pressure gradient

**Supplementary Figure-S5. Correlation matrix of FXR-related gene expression in the liver as well as serum biomarkers and indicators of disease severity.**


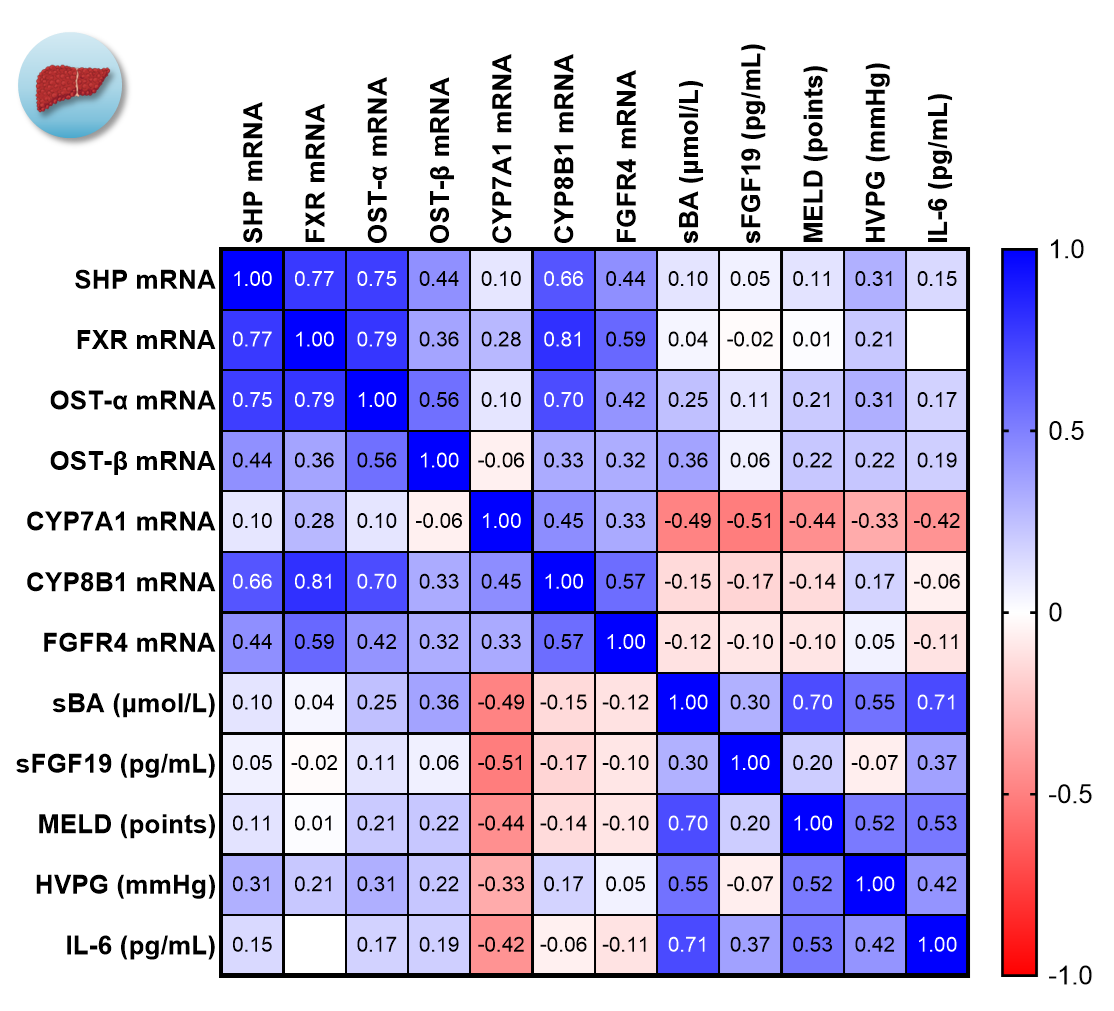


Statistical analysis: Spearman’s correlation coefficient was calculated to assess the association between continuous variables. Abbreviations: (FXR) farnesoid X receptor; (SHP) small heterodimer partner; (OST-α/-β) organic solute transporter subunit alpha/beta; ([s]FGF19) [serum] fibroblast growth factor-19; (FGFR4) fibroblast growth factor receptor-4; (CYP7A1) cholesterol 7 alpha-hydroxylase; (CYP8B1) sterol 12-alpha-hydroxylase; (sBA) serum bile acids; (MELD) Model of End Stage Liver Disease; (HVPG) hepatic venous pressure gradient; (IL-6) interleukin-6

**Supplementary Figure-S6. Fibroblast growth factor-19 (FGF19) and bile acid (BA) serum levels in patients with and without detectable hepatic expression of FGF19.**


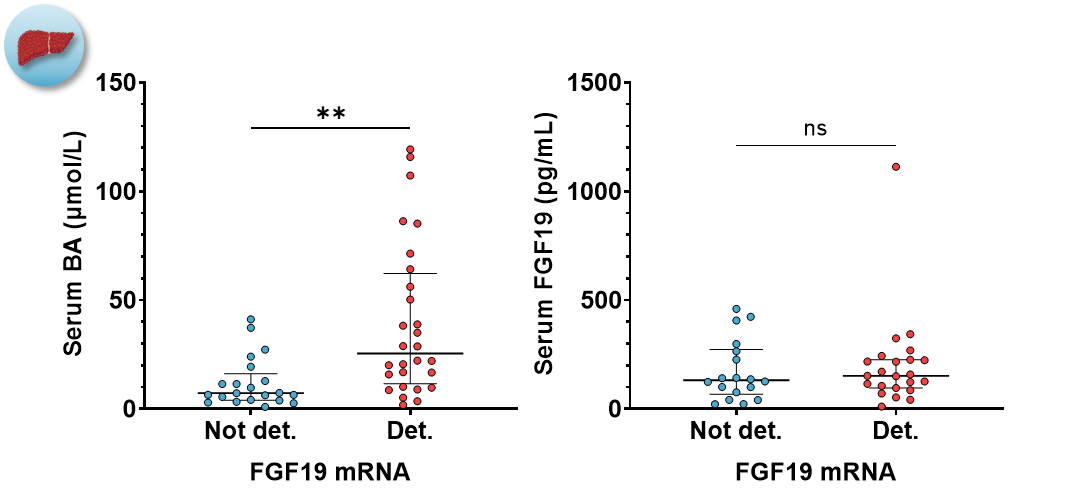


Statistical analysis: Group comparisons were performed by Mann-Whitney U test. Abbreviations: (FGF19) Fibroblast growth factor-19; (BA) bile acid; (det.) detectable

**Supplementary Figure-S7. Correlation matrix of FXR-related gene expression in the ileum as well as serum biomarkers and indicators of disease severity.**


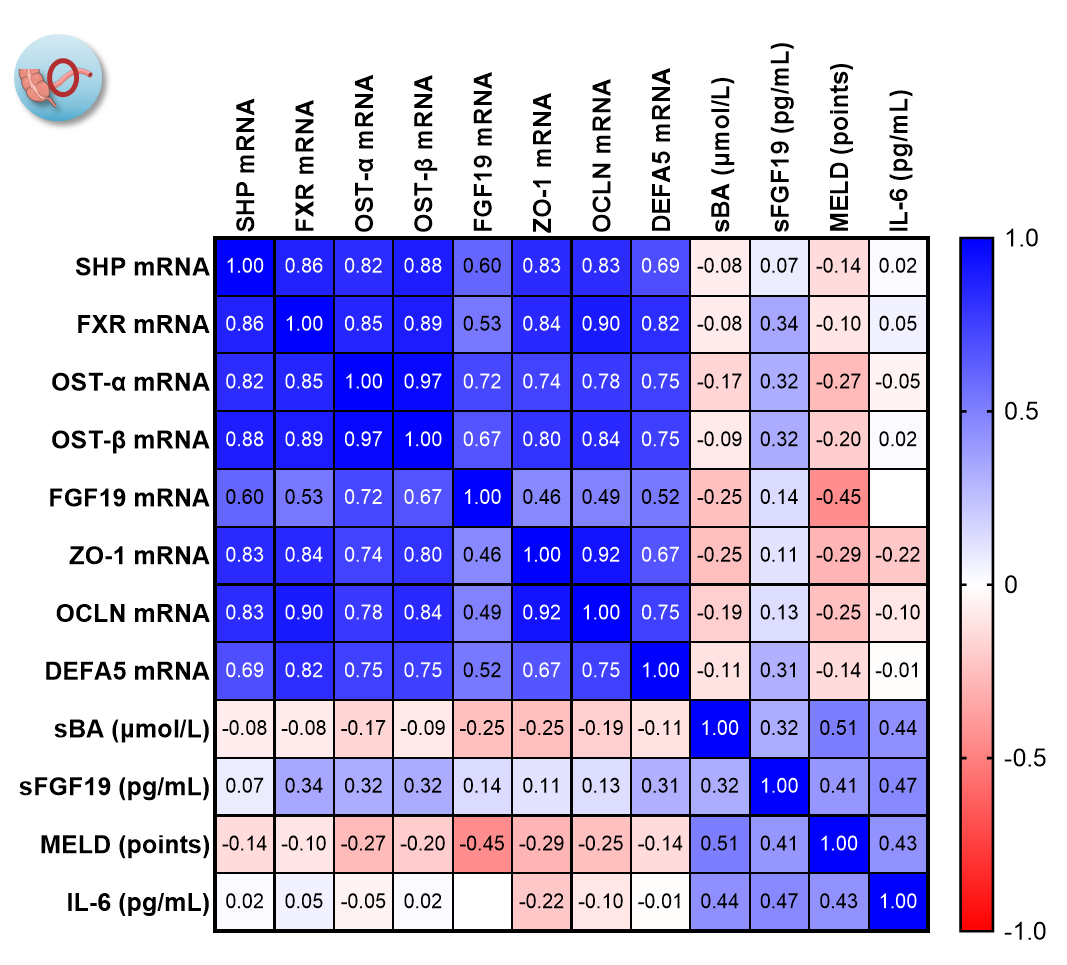


Statistical analysis: Spearman’s correlation coefficient was calculated to assess the association between continuous variables. Abbreviations: (FXR) farnesoid X receptor; (SHP) small heterodimer partner; (OST-α/-β) organic solute transporter subunit alpha/beta; ([s]FGF19) [serum] fibroblast growth factor-19; (sBA) serum bile acids; (MELD) Model of End Stage Liver Disease; (IL-6) interleukin-6; (ZO‑1) zonula occludens-1; (OCLN) occluding; (DEFA5) alpha-5-defensin

**Supplementary Figure-S8. Correlation between serum bile acids and FGF19 expression in ileum biopsies.**


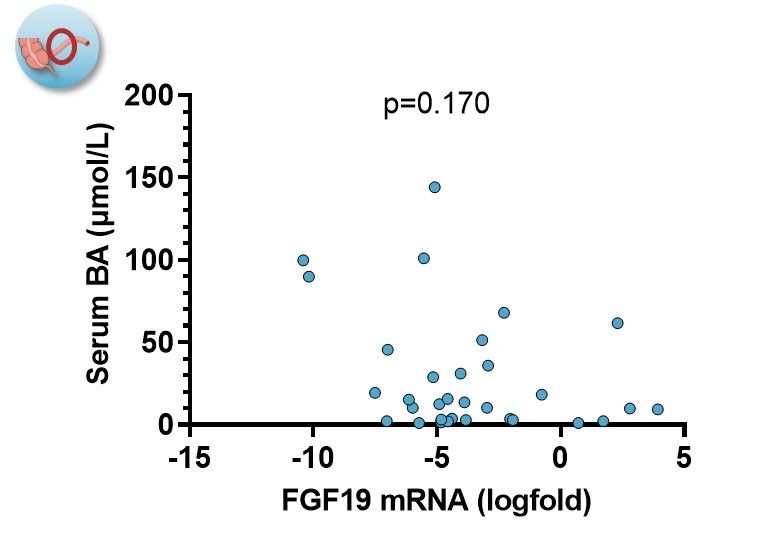


Statistical analysis: Statistical analysis: Spearman’s correlation coefficient was calculated to assess the association between continuous variables. Abbreviations: (FGF19) Fibroblast growth factor-19; (BA) bile acid

# Supplementary tables

**Supplementary Table-S1. Clinical and laboratory characteristics of patients with cACLD and dACLD undergoing HVPG measurement and liver biopsy (Cohort-Ia).**

| **Parameter** | **cACLD (n=25)** | **dACLD (n=28)** |
| --- | --- | --- |
| Age (years) | 60 (48-64) | 57 (43-64) |
| Sex (M, %) | 19 (76) | 16 (57) |
| Etiology (n, %)   - ALD - Viral - ALD + Viral - NASH - Cholestatic - Other | 7 (28) 5 (20) 1 (4) 6 (24) 1 (4) 5 (20) | 19 (68) 2 (7) 0 (0) 1 (3) 0 (0) 6 (21) |
| HVPG (mmHg) | 12 (9-15) | 17 (13-22) |
| MELD (points) | 8 (7-10) | 12 (10-16) |
| CTP-Stage   - A - B - C | 23 (92) 2 (8) 0 (0) | 7 (25) 18 (64) 3 (11) |
| BA (µmol/L) | 7.70 (3.91-21.4) | 28.8 (13.6-54.7) |
| FGF19 (pg/mL) | 126 (62.5-232) | 154 (102-239) |
| CRP (mg/dL) | 0.24 (0.12-0.55) | 0.35 (0.18-0.75) |
| IL-6 (pg/mL) | 6.02 (3.19-9.20) | 12.0 (6.30-19.7) |
| PCT (ng/mL) | 0.08 (0.07-0.12) | 0.12 (0.08-0.15) |
| LBP (µg/mL) | 7.09 (6.14-8.63) | 6.24 (4.69-7.67) |

Abbreviations: (ALD) alcohol-related liver disease; (BA) bile acid; (c/dACLD) compensated/decompensated advanced chronic liver disease; (CRP) C-reactive protein; (CTP) Child-Turcotte-Pugh; (FGF19) fibroblast growth factor-19; (HVPG) hepatic venous pressure gradient; (IL-6) interleukin-6; (LBP) lipopolysaccharide binding protein; (M) male sex; (MELD) Model of End Stage Liver Disease; (NASH) non-alcoholic steatohepatitis; (PCT) procalcitonin

**Supplementary Table-S2. Clinical and laboratory characteristics of patients with cACLD and dACLD undergoing colonoscopy with ileum biopsy (Cohort-II).**

| **Parameter** | **cACLD (n=13)** | **dACLD (n=24)** |
| --- | --- | --- |
| Age (years) | 60 (56-66) | 57 (49-62) |
| Sex (M, %) | 11 (85) | 21 (87) |
| Etiology (n, %)   - ALD - Viral - ALD + Viral - NASH - Cholestatic - Other | 6 (46) 6 (46) 0 (0) 1 (8) 0 (0) 0 (0) | 14 (5658 3 (13) 0 (0) 1 (4) 0 (0) 6 (25) |
| HVPG (mmHg)^†^ | 11 (8-15) | 19 (16-22) |
| MELD (points) | 10 (9-11) | 13 (10-14) |
| CTP-Stage   - A - B - C | 12 (92) 1 (8) 0 (0) | 6 (25) 16 (67) 2 (8) |
| BA (µmol/L) | 2.90 (2.02-10.1) | 18.2 (10.2-61.7) |
| FGF19 (pg/mL) | 67.9 (61.4-155) | 162 (60.9-319) |
| CRP (mg/dL) | 0.29 (0.17-0.67) | 0.43 (0.28-1.16) |
| IL-6 (pg/mL) | 5.27 (2.78-7.08) | 11.8 (8.71-25.2) |
| PCT (ng/mL) | 0.05 (0.02-0.09) | 0.09 (0.05-0.21) |
| LBP (µg/mL) | 7.50 (6.26-10.1) | 7.71 (5.90-12.0) |

^†^ Available in 12 (92%) patients with cACLD and 16 (67%) with dACLD.

Abbreviations: (ALD) alcohol-related liver disease; (BA) bile acid; (c/dACLD) compensated/decompensated advanced chronic liver disease; (CRP) C-reactive protein; (CTP) Child-Turcotte-Pugh; (FGF19) fibroblast growth factor-19; (HVPG) hepatic venous pressure gradient; (IL-6) interleukin-6; (LBP) lipopolysaccharide binding protein; (M) male sex; (MELD) Model of End Stage Liver Disease; (NASH) non-alcoholic steatohepatitis; (PCT) procalcitonin

**Supplementary Table-S3. Target genes and primers for RT-PCR.**

| **Name** | **Gene** | **Purpose** | **Primer serial number** | **Source** |
| --- | --- | --- | --- | --- |
| B2M | B2M | Housekeeping | Hs99999907_m1 | Thermo Fisher Scientific |
| FXR | NR1H4 | Target | Hs01026590_m1 | Thermo Fisher Scientific |
| SHP | NR0B2 | Target | Hs00222677_m1 | Thermo Fisher Scientific |
| OST-α | SLC51A | Target | Hs00380895_m1 | Thermo Fisher Scientific |
| OST-β | SLC51B | Target | Hs01057182_m1 | Thermo Fisher Scientific |
| FGF19 | FGF19 | Target | Hs00192780_m1 | Thermo Fisher Scientific |
| FGFR4 | FGFR4 | Target | Hs01106910_g1 | Thermo Fisher Scientific |
| CYP7A1 | CYP7A1 | Target | Hs00167982_m1 | Thermo Fisher Scientific |
| CYP8B1 | CYP8B1 | Target | Hs00244754_s1 | Thermo Fisher Scientific |
| ZO-1 | TJP1 | Target | Hs01551861_m1 | Thermo Fisher Scientific |
| OCLN | OCLN | Target | Hs00170162_m1 | Thermo Fisher Scientific |
| DEFA5 | DEFA5 | Target | Hs00360716_m1 | Thermo Fisher Scientific |

Abbreviations: (B2M) beta-2-microglobulin; (FXR) farnesoid X receptor; (SHP) small heterodimer partner; (OST-α/-β) Organic solute transporter subunit alpha/beta; (FGF19) fibroblast growth factor-19; (FGFR4) fibroblast growth factor receptor-4; (CYP7A1) cholesterol 7 alpha-hydroxylase; (CYP8B1) sterol 12-alpha-hydroxylase; (ZO‑1) zonula occludens-1; (OCLN) occluding; (DEFA5) alpha-5-defensin

**Supplementary Table-S4. Regulation of FXR signalling in liver and ileum biopsies of patients with ACLD.**

| **Liver** | **CON** | **cACLD** | | **dACLD** | | **ANOVA  p-value** |
| --- | --- | --- | --- | --- | --- | --- |
| FXR | 0.00±0.42 | -1.49±0.29* | **↓** | -0.61±0.21^†^ | **↔** | 0.013 |
| SHP | 0.00±0.67 | -2.66±0.29* | **↓** | -1.41±0.16*^†^ | **↓** | <0.001 |
| OST-α | 0.00±0.58 | -2.44±0.23* | **↓** | -1.16±0.20^†^ | **↔** | <0.001 |
| OST-β | 0.00±0.56 | 0.72±0.31 | **↔** | 1.97±0.31^§†^ | **↑** | 0.006 |
| FGFR4 | 0.00±0.40 | -0.55±0.17 | **↔** | -0.20±0.13 | **↔** | 0.156 |
| CYP7A1 | 0.00±1.03 | 1.56±0.64 | **↔** | 1.44±0.46 | **↔** | 0.514 |
| CYP8B1 | 0.00±0.52 | -2.04±0.20* | **↓** | -1.55±0.19* | **↓** | <0.001 |
| **Ileum** | **CON** | **cACLD** | | **dACLD** | |  |
| FXR | 0.00±0.25 | -2.14±0.46* | **↓** | -2.49±0.35* | **↓** | 0.006 |
| SHP | 0.00±0.36 | -1.77±0.72 | **↔** | -1.97±0.40^§^ | **↓** | 0.124 |
| OST-α | 0.00±0.68 | -3.16±0.50* | **↓** | -4.12±0.42* | **↓** | <0.001 |
| OST-β | 0.00±0.61 | -3.21 ±0.42* | **↓** | -3.91 ±0.40* | **↓** | <0.001 |
| FGF19 | 0.00±0.51 | -2.81±1.00 | **↔** | -4.26±0.67* | **↓** | 0.026 |
| ZO-1 | 0.00±0.24 | -0.75±0.41 | **↔** | -1.41±0.32^§^ | **↓** | 0.091 |
| OCLN | 0.00±0.15 | -1.35±0.40 | **↔** | -1.96±0.36* | **↓** | 0.030 |
| DEFA5 | 0.00±0.49 | -3.25±0.88* | **↓** | -3.11±0.38* | **↓** | 0.012 |

Figure legend: * p<0.05 vs. controls; § p≤0.10 vs. controls (statistical trend); † p<0.05 vs. cACLD. Gene expression is presented as the logfold expression (mean±SEM) as compared to the control group. Arrows represent the interpretation of gene regulation as compared to the control group (↔ equivocal, ↑ upregulation, ↓ downregulation). Statistical analysis: Ordinary one-way analysis of variance (ANOVA) with Tukey’s multiple comparisons test was applied to compare continuous variables between groups. Abbreviations: (CON) control group; (c/dACLD) compensated/decompensated advanced chronic liver disease; (FXR) farnesoid X receptor; (SHP) small heterodimer partner; (OST-α/-β) Organic solute transporter subunit alpha/beta; (FGF19) fibroblast growth factor-19; (FGFR4) fibroblast growth factor receptor-4; (CYP7A1) cholesterol 7 alpha-hydroxylase; (CYP8B1) sterol 12-alpha-hydroxylase; (ZO‑1) zonula occludens-1; (OCLN) occluding; (DEFA5) alpha-5-defensin

**Supplementary Table-S5. Missing values in patients with liver or ileum biopsy.**

| **Name** | **Readout** | **Cohort-Ia (n=53)** | | **Cohort-II (n=37)** | |
| --- | --- | --- | --- | --- | --- |
|  |  | **Missing value (n)** | **Not detectable (n)** | **Missing value (n)** | **Not detectable (n)** |
| FXR | PCR | 4 | 0 | 0 | 0 |
| SHP | PCR | 0 | 0 | 0 | 0 |
| OST-α | PCR | 0 | 0 | 0 | 0 |
| OST-β | PCR | 0 | 0 | 0 | 0 |
| FGF19 | PCR | 4 | 21 | 0 | 3 |
| FGFR4 | PCR | 0 | 0 | N/A | N/A |
| CYP7A1 | PCR | 0 | 0 | N/A | N/A |
| CYP8B1 | PCR | 0 | 0 | N/A | N/A |
| ZO-1 | PCR | N/A | N/A | 0 | 0 |
| OCCL | PCR | N/A | N/A | 0 | 0 |
| DEFA5 | PCR | N/A | N/A | 0 | 0 |
| Total BA (µmol/L) | Laboratory | 0 | 0 | 2 | 0 |
| FGF19 (pg/mL) | Laboratory | 8 | 0 | 9 | 0 |
| CRP (mg/dL) | Laboratory | 0 | 0 | 0 | 0 |
| IL-6 (pg/mL) | Laboratory | 0 | 0 | 1 | 0 |
| PCT (ng/mL) | Laboratory | 0 | 0 | 1 | 0 |
| LBP (µg/mL) | Laboratory | 0 | 0 | 2 | 0 |

Abbreviations: (FXR) farnesoid X receptor; (SHP) small heterodimer partner; (OST-α/-β) Organic solute transporter subunit alpha/beta; (FGF19) fibroblast grwoth factor-19; (FGFR4) fibroblast growth factor receptor-4; (CYP7A1) cholesterol 7 alpha-hydroxylase; (CYP8B1) sterol 12-alpha-hydroxylase; (ZO‑1) zonula occludens-1; (OCLN) occludin; (DEFA5) alpha-5-defensin; (IL-6) interleukin-6; (BA) bile acid; (WBC) white blood cell; (CRP) C-reactive protein; (IL-6) interleukin-6

# References (used in the supplementary material)

1. Reiberger, T., et al., *Measurement of the Hepatic Venous Pressure Gradient and Transjugular Liver Biopsy.* J Vis Exp, 2020(160).

2. Livak, K.J. and T.D. Schmittgen, *Analysis of relative gene expression data using real-time quantitative PCR and the 2(-Delta Delta C(T)) Method.* Methods, 2001. **25**(4): p. 402-8.
